# Relationship of phasic left atrial volume and emptying function to left ventricular filling pressure: a cardiovascular magnetic resonance study

**DOI:** 10.1186/1532-429X-15-99

**Published:** 2013-10-29

**Authors:** Kanna Posina, Jeannette McLaughlin, Peter Rhee, Laura Li, Joshua Cheng, William Schapiro, Ronald J Gulotta, Andrew D Berke, George A Petrossian, Nathaniel Reichek, Jie J Cao

**Affiliations:** 1 St. Francis Hospital, Roslyn, NY, USA; 2 State University of New York, Stony Brook, 100 Port Washington Boulevard, Roslyn, NY, 11576, USA

**Keywords:** Left atrial function, Left atrial volume, Left ventricular filling pressure, Cardiovascular magnetic resonance

## Abstract

**Background:**

Left atrial volume (LAV) and emptying fraction (LAEF) are phasic during cardiac cycle. Their relationships to left ventricular end diastolic pressure (LVEDP) have not been fully defined.

**Methods:**

Forty one patients undergoing clinically indicated left heart catheterization were recruited for same day cardiovascular magnetic resonance (CMR). LAV and LAEF were assessed in cine images using biplane area and length method. Three phasic LAV was assessed at LV end systole (LAV_max_), LV end diastole (LAV_min_) and late LV diastole prior to LA contraction (LAV_ac_). LAEF was assessed as global LAEF (LAEF_Total_), passive (LAEF_Passive_) and active LAEF (LAEF_Contractile_). The relationships of phasic LAV and LAEF to LVEDP were assessed using Receiver operating characteristic comparing areas under the curves (AUC).

**Results:**

The mean age of the patients was 59 years. A history of heart failure was present in 16 (39%) with NYHA functional class III or IV in 8 (20%) patients. Average LV ejection fraction was 49 ± 16% ranging from 10% to 74% and LVEDP by catheterization 14 ± 8 mmHg ranging from 4 mmHg to 32 mmHg. LAV_min_ had the strongest association with LVEDP elevation (>12 mmHg) (AUC 0.765, p = 0.002), as compared to LAV_max_ (AUC 0.677, p = 0.074) and LAV_ac_ (AUC 0.735, p = 0.008). Among three phasic LAEF assessed, LAEF_Total_ had the closest association with LVEDP elevation (AUC 0.780, p = 0.001), followed by LAEF_Contractile_ (AUC 0.698, p = 0.022) and LAEF_Passive_ (AUC 0.656, p = 0.077).

**Conclusions:**

Increased LAV_min_ and decreased LAEF_Total_ have the best performance in identifying elevated LVEDP among three phasic LAV and LAEF analyzed. Future studies should further characterize LA phasic indices in clinical outcomes.

## Background

Left atrial (LA) dilatation is found in many conditions, including atrial fibrillation, left ventricular (LV) systolic and diastolic dysfunction, congestive heart failure, and valvular heart disease [1-4]. Increased maximal LA size measured at LV end systole has been associated with cardiovascular morbidity and mortality in population-based studies, a finding attributed to the detrimental effect of chronically increased LV filling pressure which results in LA remodeling over time [3,5-8]. LA function has three distinct phases during cardiac cycle: a filling phase during ventricular systole, a conduit phase during early diastolic rapid ventricular filling and an active contraction phase during late diastole [9]. Early reports based on echocardiographic data demonstrated a direct relationship of increased filling pressure to increased LA size and reduced LA function [10]. However, the relationship of LA phasic volume and function to LV filling pressure have not been fully defined.

The standard method of determining phasic LA volumes (LAV) is based on a time-volume curve depicting LAV over the entire cardiac cycle. A simpler alternative, which is commonly used in clinical studies, assesses LAV in single-phase analyses characterized by mitral valve position [11-13]. But it is unclear whether the single-phase method provides results comparable to those obtained using the more laborious volume curve approach.

We sought to assess the relationship of phasic LAV and LA function to LV filling pressure and to compare the single-phase LAV method to the standard multi-phase method using cardiovascular magnetic resonance (CMR) in a group of patients who underwent clinically indicated right and left heart catheterization.

## Methods

### Study population

The study protocol was approved by the St. Francis Hospital Institutional Review Board. All subjects were prospectively recruited with written consent. There were 41 patients undergoing clinically indicated right and left heart catheterization. Exclusions included atrial fibrillation, impaired renal function with glomerular filtration rate < 45 mL/min/1.73 m^2^; claustrophobia; pacemaker/defibrillator implantation; or other metallic hazards.

### Hemodynamics

LV end diastolic pressure (LVEDP) was obtained during left heart catheterization following standard clinical protocol. Hemodynamic tracings obtained during quiet respiration were recorded and stored electronically. Two experienced cardiologists reviewed tracings and the values of LVEDP were determined by consensus. To match the inspiration breath-hold during CMR image acquisition LVEDP at inspiration was used.

### CMR

All subjects underwent CMR in a 1.5 T Avanto scanner (Siemens) using 8-element phased array surface coil. Steady-state free precession (SSFP) cine images were acquired during inspiratory breath-holds with retrospective ECG gating in long axis 2-, 3- and 4-chamber views. A stack of 8-mm thick short axis images was obtained with 2-mm gaps. The average field of view was 240 mm, echo time 1.3 ms, repetition time 3.1 ms, flip angle 70°, matrix 192 × 154 yielding an in-plane resolution of 1.6 × 1.3 mm and temporal resolution of 30–40 ms. The average number of phases was 20 to 30, varying by heart rate to reach consistent temporal resolution.

### Image analysis

**Figure 1:**
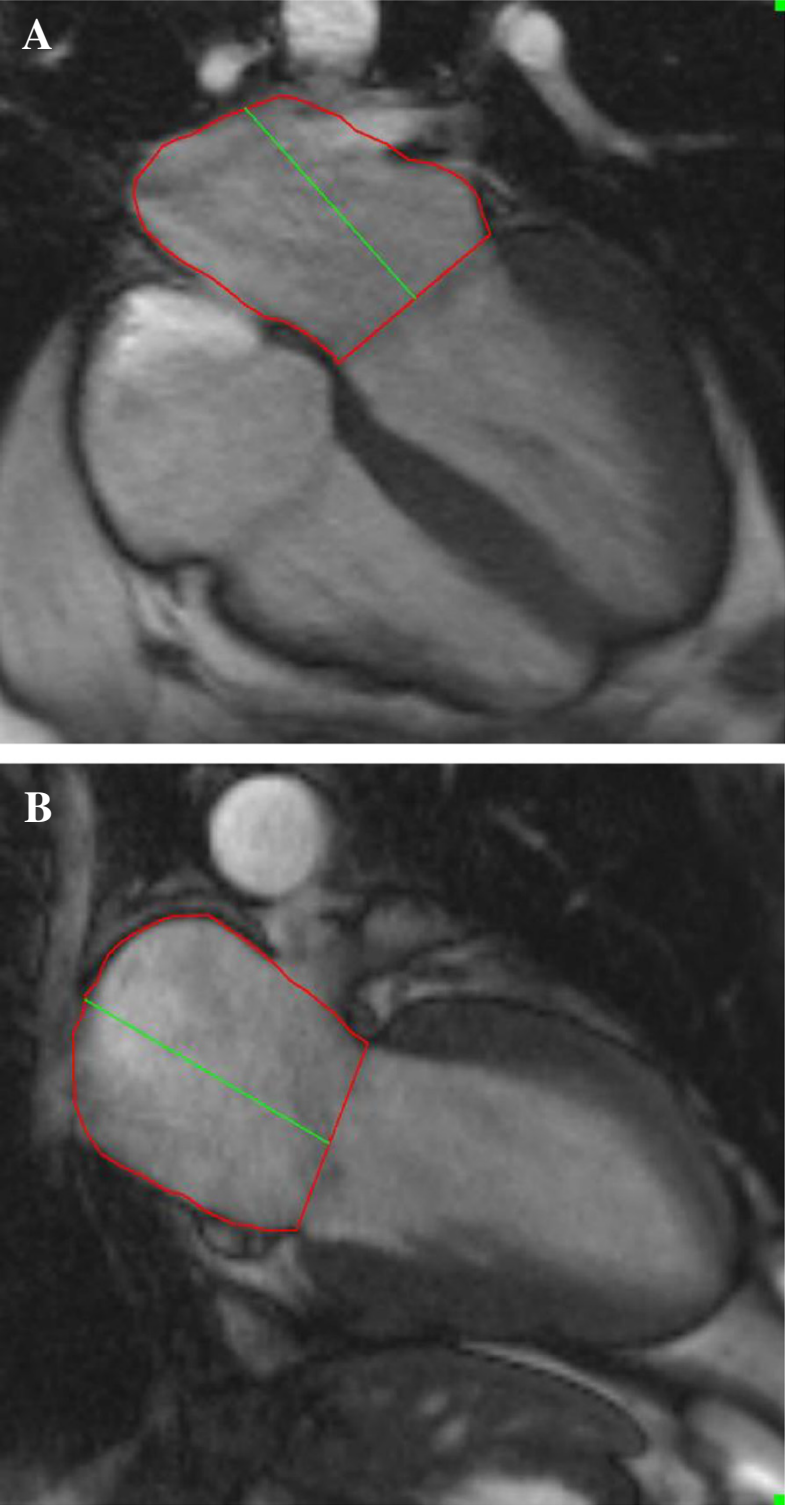
Left atrial area and length assessment using the 4 (A) and 2 (B) chamber views at left ventricular end systole.

**Figure 2:**
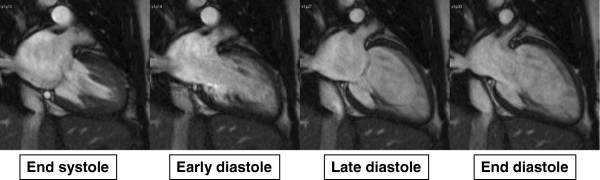
**Illustrations of left atrial phasic changes on MR cine images during cardiac cycle when atrial size is at the largest during end systole and smallest during end diastole.** The left atrial precontractile phase is determined at the late diastole when mitral valve is nearly closed before it opens again after atrial contraction.

**Figure 3:**
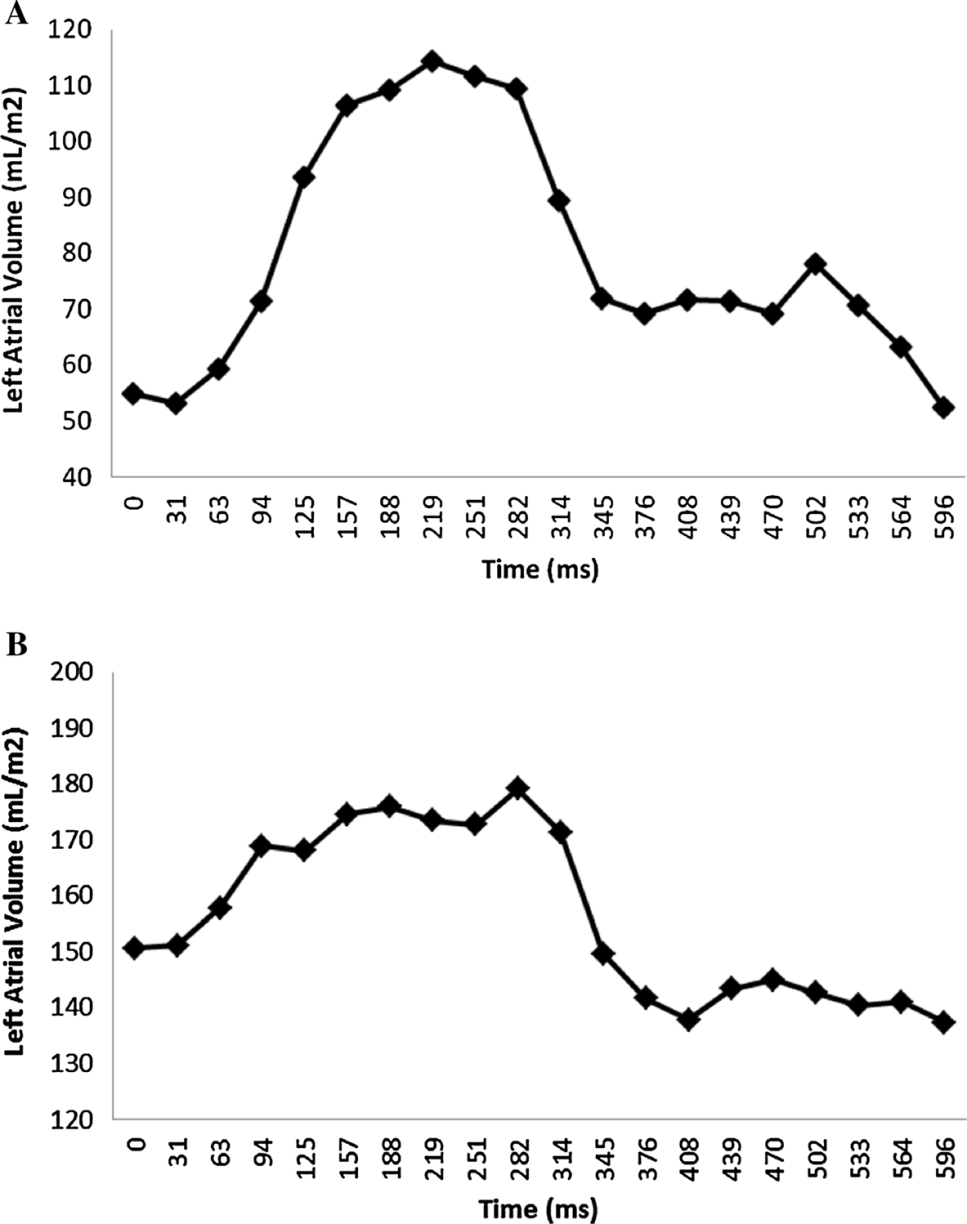
Examples of time volume curves for patient with normal global left atrial emptying function (58%) and left ventricular end diastolic pressure (7 mmHg) (A) and for patient with reduced global left atrial emptying function (23%) and elevated left ventricular end diastolic pressure (32 mmHg) (B).

Volumetric short axis cine images were analyzed using commercially available software (QMass by Medis, Netherland). Left and right ventricular volumes, ejection fraction and myocardial mass were assessed and normalized to body surface area. LAV were calculated using a biplane area and length method following the formula: 0.85×A1×A2/L, where A1 and A2 were areas measured by planimetry in 2- and 4-chamber views, respectively and L was the length of LA perpendicular to the center of mitral annulus in the 4-chamber plane (Figure 1) [13]. Due to inconsistent presence of the LA appendage in the 2 chamber view we excluded LA appendage from all analyses. LAV was assessed at LV end systole (LAV_max_), at LV late diastole just before LA contraction (LAV_ac_) and LV end diastole (LAV_min_) (Figure 2) [8,12]. LAV was determined using both single-phase analysis and multi-phase time-volume curves. For the single-phase method, all slices of the 2- and 4-chamber cines were reviewed and LAV_max_, LAV_ac_, and LAV_min_ assessed at time points just before mitral valve opening, immediately prior to atrial contraction and at the time of mitral valve closure_,_ respectively. Using the multi-phase time-volume curve method data points for LAV_max_, LAV_ac_ and LAV_min_ were defined as the maximum volume during systole, maximum volume prior to atrial contraction and minimum volume at the end of diastole respectively (Figure 3). Inter and intra-observer variability of LAV quantitation was determined in five randomly selected cases for both methods. The intra and inter-observer concordance correlation coefficients were 0.99 and 0.98 for the single-phase method, and 0.92 and 0.90 for multi-phase method, respectively. Global emptying function (LAEF_Total_), passive LAEF (LAEF_Passive_) and active LAEF (LAEF_Contractile_) were defined as fractional volume changes as follows. LAEF_Total_ = (LAV_max_-LAV_min_)/LAV_max_, LAEF_Passive_ = (LAV_max_-LAV_ac_)/LAV_max_ and LAEF_Contractile_ = (LAV_ac_-LAV_min_)/LAV_max_, respectively [8].

### Statistical analysis

Categorical variables were expressed as proportions and continuous variables as means ± standard deviations (SD). Categorical variables were compared using Fisher’s exact test and continuous variables using Student’s *t* test. Receiver operating characteristic analysis was performed to evaluate the associations of LAV and LA function variables with LVEDP and areas under the curves compared. Sensitivity testing based on the optimal sensitivity and specificity in Receiver operating characteristic analysis was used to determine the cut points of phasic LAV and LA function in identifying associated elevated LVEDP (> 12 mmHg). Bland-Altman plots were used to compare LAV measurements derived using single-phase and multi-phase methods. For all statistical analyses, p < 0.05 was considered significant. Statistical analysis was performed using MedCalc 11.3 (MedCalc Software, Belgium) and SPSS 17 (SPSS Inc, Chicago, IL).

## Results

The mean age of the patients was 59 years. A history of coronary artery disease was present in 27% of patients and history of congestive heart failure in 39%. NYHA functional class III or IV was present in 20% of patients. Average LV ejection fraction was 49 ± 16% ranging from 10% to 74% with 32% of patients having LV ejection fraction < 50%. Mean LVEDP was 14 ± 8 mmHg ranging from 4 mmHg to 32 mmHg. The most prevalent significant valvular dysfunction (≥2+) was aortic insufficiency (17%) followed by mitral regurgitation (12%) (Table 1).

**Table 1:** Patient characteristics (N = 41)

	**Mean ± SD or N (%)**
**Demographics**	
Age (years)	59 ± 15
Female	15 (37)
Body mass index (kg/m^2^)	30 ± 6
Hypertension	26 (63)
Hyperlipidemia	24 (59)
Diabetes	7 (17)
History of coronary artery disease	11 (27)
History of heart failure	16 (39)
NYHA functional class III or IV	9 (20)
**Hemodynamics**	
Heart rate (beats/sec)	70 ± 14
Systolic pressure (mmHg)	127 ± 16
Diastolic pressure (mmHg)	75 ± 12
LVEDP (mmHg)	14 ± 8
**CMR indices**	
LV end diastolic volume (mL/m^2^)	90 ± 39
LV end systolic volume (mL/m^2^)	55 ± 59
LV ejection fraction (%)	49 ± 16
LV Mass Index (g/m^2^)	68 ± 29
RV end diastolic volume (mL/m^2^)	68 ± 21
RV end systolic volume (mL/m^2^)	35 ± 21
RV ejection fraction (%)	52 ± 14
RV Mass Index (g/m^2^)	19 ± 6
**Significant valvular dysfunction (≥2+)**	
Aortic stenosis	3 (7)
Aortic regurgitation	7 (17)
Mitral regurgitation	5 (12)
Tricuspid regurgitation	1 (2)
Pulmonic regurgitation	1 (2)

**Figure 4:**
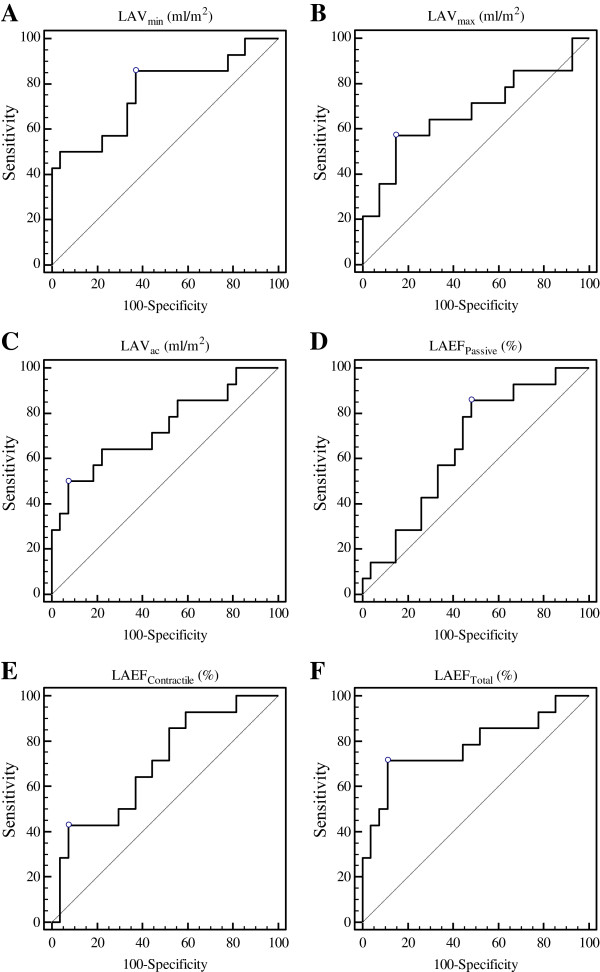
**Area under the curves by receiver operator characteristic analysis assessing the relationships of the following left atrial indices to elevated left ventricular end diastolic pressure (> = 12 mmHg): (A), left atrial volume at left ventricular end diastole (LAV**_**min**_**); (B), left atrial volume at left ventricular end systole (LAV**_**max**_**); (C), left atrial volume at left ventricular late diastole before left atrial systole (LAV**_**ac**_**); (D), passive left atrial emptying function (LAEF**_**Passive**_**); (E), active left atrial emptying function (LAEF**_**Contractile**_**); and (F), global left atrial emptying function (LAEF**_**Total**_**).**

LAV_max_, LAV_ac_ and LAV_min_ were strongly correlated with each other (r = 0.938 between LAV_max_ and LAV_ac_, r = 0.953, between LAV_min_ and LAV_ac_, r = 0.896 between LAV_max_ and LAV_min_, all p < 0.001). Each was significantly larger in subjects with elevated LVEDP (>12 mmHg) than in those with normal LVEDP (≤12 mmHg) (Table 2). Pearson correlation of LAV to LVEDP was modest with correlation coefficient of 0.536 (p < 0.001), 0.412 (p = 0.007), 0.482 (p = 0.001) for LAV_min_, LAV_max_ and LAV_ac_, respectively. Using ROC analyses, the association with elevated LVEDP was stronger for LAV_min_ and LAV_ac_ than for LAV_max_ (Figure 4A-C). In the sensitivity testing, LAV_min_ was more sensitive, while LAV_max_ and LAV_ac_ were more specific in identifying elevated LVEDP (Table 3). The three LAEF indices correlated with each other moderately (r = 0.621 between LAEF_Total_ and LAEF_Passive_, r = 0.629 between LAEF_Total_ and LAEF_Contractile_, r = 0.581 between LAEF_Contractile_ and LAEF_Passive_, all p < 0.001). Similar to phasic LAV, all three phasic LAEF indices were lower in subjects with elevated LVEDP than in those without (Table 2). Using ROC analysis LAEF_Total_ had the strongest association with elevated LVEDP followed by LAEF_Contractile_ and LAEF_Passive_ (Figure 4D-F). In the sensitivity testing, LAEF_Total_ and LAEF_Contractile_ were more specific in predicting elevated LVEDP while LAEF_Passive_ was more sensitive (Table 3).

**Table 2:** Comparisons of LA Indices between subjects with elevated (>12 mmHg) and normal LVEDP (≤12 mmHg)

	**LVEDP (>12 mmHg) (N = 14)**	**LVEDP (≤12 mmHg) (N = 27)**	**p-value**
LAV_min_ (mL/m^2^)	35 ± 20	22 ± 9	0.016
LAV_max_ (mL/m^2^)	56 ± 21	44 ± 13	0.065
LAV_ac_ (mL/m^2^)	48 ± 18	37 ± 11	0.027
LAEF_Passive_ (%)	16 ± 8	16 ± 8	0.078
LAEF _Contractile_ (%)	25 ± 12	35 ± 9	0.029
LAEF_Total_ (%)	41 ± 16	51 ± 10	0.005
LVEDP (mmHg)	22 ± 7	9 ± 2	<0.001

**Table 3:** ROC analysis assessing association of LA indices to increased LVEDP (>12 mmHg) using single-phase method

	**AUC**	**p-value**	**Criterion cutoff value**	**Sensitivity**	**Specificity**
LAV_min_	0.765	0.002	≥ 23 ml/m^2^	86%	63%
LAV_max_	0.677	0.074	≥ 54 ml/m^2^	57%	85%
LAV_ac_	0.735	0.008	≥ 47 ml/m^2^	50%	91%
LAEF_Passive_	0.656	0.077	≤ 17%	86%	52%
LAEF_Contractile_	0.698	0.022	≤ 16%	43%	93%
LAEF_Total_	0.780	0.001	≤ 35%	71%	89%

**Figure 5:**
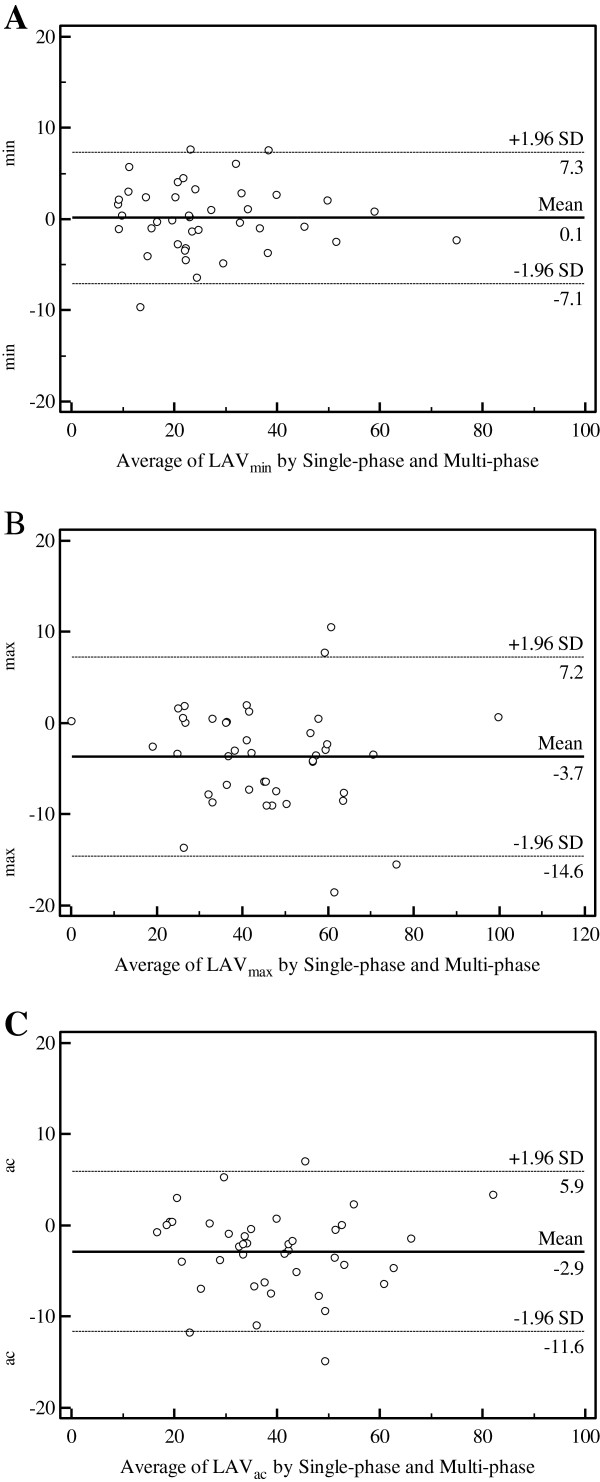
**Comparisons of single-phase and multi-phase methods using Bland-Altman plots comparing single-phase and multi-phase methods in the assessment of left atrial volume at left ventricular end diastole (LAV**_**min**_**) (A), left atrial volume at left ventricular end systole (LAV**_**max**_**) (B); and left atrial volume at left ventricular late diastole before left atrial systole (LAV**_**ac**_**) (C).**

LAV from time-volume curves demonstrated the expected large phasic volume changes in normal subjects (Figure 3A), but the relative volume change was much smaller in patients with elevated LVEDP and large atrial volumes (Figure 3B). Bland-Altman plots showed small mean differences between single-phase and multi-phase volumes of −3.7 ± 10.9 ml/m^2^, 0.1 ± 7.2 ml/m^2^ and −2.9 ± 8.8 ml/m^2^ for LAV_max_, LAV_min_ and LAV_ac_, respectively (Figure 5).

## Discussion

In this study we demonstrated that increased LA volumes at three different cardiac cycle phases were all associated with increased LVEDP. While LAV_min_ was most sensitive to increased LVEDP, LAV_max_ and LAV_ac_ were more specific correlates of elevated LV filling pressure. Overall, LAV_min_ performed the best in stratifying LVEDP in ROC analysis. Of the three LA functional indices, LAEF_total_ was the best measurement to identify elevated LVEDP. LAEF_Passive_ was the most sensitive while LAEF_total_ and LAEF_contractile_ were more specific in stratifying elevated LVEDP. We also found that single-phase quantitation is a good alternative to the more laborious multi-phase quantitation method.

End systolic LAV_max_ has been shown previously to be a useful marker of LV diastolic function and a robust index of clinical outcome risk including atrial fibrillation, stroke, and heart failure [1-3,5,14-16]. One likely mechanism is LA remodeling in response to increased LV filling pressure [9]. Therefore a cross-sectional evaluation of LA size is viewed as a marker of LV filling pressure over time. Most published epidemiologic and clinical studies rely on maximal LA volume at LV end systole (LAV_max_) [13,14,17-19]. But a recent clinical study exploring the relationship of LA size at different cardiac cycle phases to clinical outcomes suggested that LAV_min_ taken at LV end diastole is superior to LAV_max_ as a marker of clinical outcome risk [20]. Few studies have comprehensively assessed the relationship of phasic LA volumes and phasic LA function to LV filling pressures.

With the growing interest in LA assessment in clinical and epidemiological studies it is important to delineate the pros and cons of alternative evaluation methods [7,9]. The multi-phase method, which is considered to be the standard approach, is cumbersome and time-consuming [11,12]. Furthermore, it can be difficult to identify LA phases from the time-volume curve when there is limited LA volume change in subjects with large LA volumes and poor LA function. The single-phase method, using 3 phases based on mitral valve position is widely used in clinical studies because of its simplicity [13]. However, published validation data is limited. We demonstrated that differences in LA volumes between two methods were small, while reproducibility of the single-phase method was excellent, supporting the use of single-phase approach. However, we acknowledge that our findings are limited to biplane-length approach only and can not be generalized to other method of LAV assessment.

There are limitations to our study. As expected the chronicity of increased LV filling pressures is a variable which may influence the extent of LA remodeling and LA active contraction and could not be easily determined in this clinical population. Mitral regurgitation was present in some patients and can further complicate the relationship of LA size and function to LV filling pressure. While all participants were reportedly stable small variation in hemodynamics are inevitable given that CMR was performed within 5 hours of catheterization. Contrast or sedatives commonly given during left heart catheterization may reduce LV filling pressure rendering a possible underestimation of the relationship of the LA indices with LVEDP. We also assessed LAV using the biplane area length method rather than the gold standard Simpson’s rule method. However, the biplane method has been well validated and is of the most relevant approach because of its speed, simplicity and widespread use in both clinical and epidemiological studies.

## Conclusions

In conclusion, increased LA volumes at multiple time points in the cardiac cycle and decreased LA functional indices were all associated with increases in LVEDP. Among them increased LAV_min_ and decreased LAEF_Total_ were most closely associated with elevated LVEDP in ROC analysis. Single-phase LA volume and function indices are good alternatives to the multi-phase volume curve methods.

## Abbreviations

CMR: Cardiovascular magnetic resonance; LV: Left ventricle; RV: Right ventricle; LVEDP: Left ventricular End Diastolic Pressure; LA: Left atrium; LAV: Left atrial volume; LAVmax: Left atrial volume at left ventricular end systole; LAVmin: Left atrial volume at left ventricular end diastole; LAVac: Left atrial volume at left ventricular late diastole before left atrial contraction; LAEF: Left atrial emptying function; LAEFTotal: Global left atrial emptying fraction; LAEFPassive: Passive left atrial emptying fraction; LAEFContractile: Active left atrial emptying fraction; ROC: Receiver operating characteristics.

## Competing interests

The authors declare that they have no competing interests.

## Authors’ contributions

KP, Analysis and interpretation of the data, image analysis, and drafted manuscript. JM, Patient recruitment and clinical data management. PR, Patient recruitment and clinical data collection. LL, Image analysis. JC, Image acquisition. WS, Image acquisition. RG, Hemodynamic data collection. AB, Hemodynamic data collection. GP, Hemodynamic data collection. NR, Critical review of the manuscript. JJC, Conception and design of study, data review and analysis, critical review of the manuscript, and final approval of submission. All authors read and approved the final manuscript.
